# Jejunal microbiota of broilers fed varying levels of mineral phosphorus

**DOI:** 10.1016/j.psj.2023.103096

**Published:** 2023-09-09

**Authors:** Adewunmi O. Omotoso, Henry Reyer, Michael Oster, Siriluck Ponsuksili, Klaus Wimmers

**Affiliations:** ⁎ Research Institute for Farm Animal Biology (FBN), 18196 Dummerstorf, Germany; † Faculty of Agricultural and Environmental Sciences, Justus-von-Liebig-Weg 6b, University of Rostock, 18059 Rostock, Germany

**Keywords:** phosphorus excretion, intestinal microbiota, mineral supply, mineral homeostasis, nutritional conditioning

## Abstract

Efforts to achieve sustainable phosphorus (**P**) inputs in broiler farming which meet the physiological demand of animals include nutritional intervention strategies that have the potential to modulate and utilize endogenous and microbiota-associated capacities. A temporal P conditioning strategy in broiler nutrition is promising as it induces endocrinal and transcriptional responses to maintain mineral homeostasis. In this context, the current study aims to evaluate the composition of the jejunal microbiota as a functional entity located at the main absorption site involved in nutrient metabolism. Starting from a medium or high P supply in the first weeks of life of broilers, a depletion strategy was applied at growth intervals from d 17 to 24 and d 25 to 37 to investigate the consequences on the composition of the jejunal microbiota. The results on fecal mineral P, calcium (**Ca**), and phytate contents showed that the diets applied to the depleted and non-depleted cohorts were effective. Microbial diversity in jejunum was represented by alpha diversity indices which appeared unaffected between dietary groups. However, chickens assigned to the dietary P depletion groups showed significantly higher abundances of *Facklamia, Lachnospiraceae*, and *Ruminococcaceae* compared to non-depleted control groups. Based on current knowledge of microbial function, these microorganisms make only a minor contribution to the birds' adaptive mechanism in the jejunum following P depletion. Microbial taxa such as *Brevibacterium, Brachybacterium*, and genera of the *Staphylococcaceae* family proliferated in a P-enriched environment and might be considered biomarkers for excessive P supply in commercial broiler chickens.

## INTRODUCTION

Dietary phosphorous (**P**) is essential to all life forms owing to its multifaceted functions within the organismal biosystem. In the broiler, the significance of P as a mineral constituent of the diet has been established due to its pivotal role in physiological processes relating to the bird's growth and productivity when efficiently utilized. In plant-based diets, P is stored as the salt form of phytic acid [*myo*-inositol 1,2,3,4,5,6-hexakisphosphate; InsP_6_], also referred to as phytate. However, broilers have limited capacity to utilize phytate-P due to inadequate production of enteral phytase/phosphatases needed to hydrolyze phytate (Rama Rao et al., 1999), accounting for environmental P losses (Panagos et al., 2022). On conventional farms, both mineral P and phytases of microbial origin are supplemented to diets. The latter is used to enzymatically degrade phytate and release inorganic P, a practice prohibited in organic livestock production (Council of the European Union, 2007). However, apart from such exogenous routes regarding the enteric bioavailability of various P-sources, the broiler chicken has demonstrated the capacity to efficiently allocate P resources through endogenous mechanisms involving mineral deposition in the bone, mediated by endocrinal and transcriptional control and adaptation in the intestine and kidney (Shao et al., 2018; Omotoso et al., 2023).

With regard to the efficient utilization of plant-bound P, the diversity and functional contribution of the intestinal microbiota is also an interesting target. The microbiota represents a dynamic constituent of the gastrointestinal tract (**GIT**) that colonizes after hatching and is defined by several factors that can be broadly divided into i) host characteristics, for example, bird age, strain, sex, GIT section, and ii) the environmental factors, for example, husbandry system, feed, geographic location, and biosecurity (Kers et al., 2018; Ngunjiri et al., 2019). Interestingly, colonization cascades have been described in which intestinal pioneer colonizers could shape the subsequent microbiota composition of individual birds (Rubio, 2019). In fact, the microbiota is acknowledged to contribute to the dynamics of complex structural, metabolic and immunological processes that define the host's health, welfare and age-appropriate development (Rubio et al., 2014).

The intestinal microbiota contains specific phosphatase-secreting microbes such as the *Bifidobacteria* (Haros et al., 2005) and isolates of *Lactobacillus* (Kim et al., 2007), which are capable of hydrolyzing phytate and release inorganic P to the host for absorption. Moreover, the dietary supply of macrominerals such as P and calcium to the monogastric species has been reported to modulate the gut microbiota (Ptak et al., 2015; Reyer et al., 2021), thus, indicating the microbiota as a potent, functional entity involved in nutrient metabolism (Grice and Segre, 2012).

Furthermore, accumulating scientific studies on the chicken microbiota focused on the distal ileocecal region of the GIT, for example, the caeca or colon, due to the high diversity of the microbial community in this GIT section crucial to mediate the hydrolysis of phytate which determines the corresponding levels of P and inositol phosphates excreted to the environment (Witzig et al., 2015; Yan et al., 2017). However, the homeostasis and metabolism of P are initiated in the proximal small intestine, specifically, the jejunum, where active and passive transport processes enable P uptake and thus facilitate increased absorption and utilization after enzymatic phytate degradation (Hurwitz and Bar, 1970). Hence, investigating the broiler's jejunal microbiota composition under different dietary P supply might be informative.

Additionally, due to P loss concerns, recent studies which investigated the broiler's physiological adaptation to moderate dietary P reductions initiated at the early growth phase highlighted the bird's capacity to incorporate different intrinsic compensatory mechanisms to define nutrient efficiency with maturity (Valable et al., 2018; Baradaran et al., 2021). Endogenous mechanisms observed include the synergy between endocrinal regulators (e.g., calcitriol), gene expression (e.g., *SLC34A2*), and the contribution of intrinsic organismal P reservoirs such as the bone.

Physiologically, calcitriol (1,25(OH)_2_ vitamin D) mediates transcellular P uptake in the intestine via its receptor (**VDR**) which acts on the promoter of the sodium-dependent P transporter to stimulate its expression. The bone facilitated P homeostasis due to its remodeling attributes (e.g., dynamics of mineral P storage and resorption), by enabling effective P resource allocation and adaptation in response to the reduced dietary P intake (Hu et al., 2018; Li et al., 2020). Moreover, previous studies in pigs fed variable dietary P levels showed significantly differential abundances of the intestinal microbial genera, suggesting the possibility of targeted manipulation of the microbial community in the intestine by feeding interventions for an improved intestinal phytate utilization (Reyer et al., 2021).

Therefore, we hypothesized that the jejunal microbiota of broilers fed varying dietary P levels from the early grower until finisher phase synergizes with the endogenous mechanisms adopted by the bird to maintain P homeostasis. The objective of the present study was to evaluate the jejunal microbiota composition of broilers subjected to P depletion throughout the grower and finisher stages. Additionally, corresponding measurements of total fecal P, calcium, and phytate were conducted to approximate the unutilized fractions.

## MATERIALS AND METHODS

### Ethical Statement

The animal experimental setup was approved by the Scientific Committee of the Research Institute for Farm Animal Biology (**FBN**) and licensed by the animal welfare and ethics committee of the state Mecklenburg-Western Pomerania, Germany (LALLF 7221.3-1-051/16).

### Experimental Birds, Management, and Diets

The feeding trial refers to a larger experiment involving previously reported performance data (Omotoso et al., 2023). The experiment was conducted at the poultry research facility of the FBN. In this study, a total of *n* = 110 Ross 308 broiler hatchlings of both sexes obtained from WIMEX Agrarprodukte GmbH was used (Regenstauf, Germany). In brief, birds were raised on wood shavings as litter material in pens of 3.8 m^2^ with a stocking density below 25 kg/m^2^, which meets current organic standards for broiler spacing requirements. Each pen was equipped with feeders and nipple drinkers for unrestricted access to feed and water. Birds were randomly allotted to 2 dietary groups comprising 55 animals each housed in 1 pen per treatment. From the grower stage at d 11, a total of 39 birds per group remained in the respective pens, while a subset of sex-balanced broiler chickens (*n* = 16 per dietary group) were transferred into individual metabolic units (45 cm × 45 cm × 45 cm) equipped with feeders and nipple drinkers to enable access to feed and water. The metabolic units were designed to ensure the birds' visual contact with their conspecifics and guarantee to record individual bird's zootechnical parameters including feed intake. The dietary regimen comprised starter (d 1–10), grower (d 11–24), and finisher diets (d 25–37). The wheat-corn-soybean meal-based diet was formulated without the addition of nonstarch polysaccharide enzymes or phytase. The fed diets for starter, grower, and finishers were pelleted and formulated according to the nutrient recommendations of the Gesellschaft für Ernährungsphysiologie (GFE, 1999) except for P (Supplemental Table 1). The experimental diets contained either recommended (M; 100% according to Ross, 2014) or higher (H; +50%) amounts of nonphytate P (**nPP**) in the respective grower and finisher feeds. From d 17, half of the broilers in cages were subjected to dietary P depletion (−50%), wherein chickens were offered lowered dietary P levels in the grower stage (i.e., ML, HL) and finisher phases (MLL, HLL). Accordingly, the experimental design also included the non-depleted (recommended and high) groups for the respective stages, that is, MM and HH for grower and MMM and HHH for the finisher phases, respectively. As previously published (Omotoso et al., 2023), the analyzed values for crude protein (**CP**) in grower diets (L: 225 g/kg; M: 217 g/kg; H: 221 g/kg) and finisher diets (L: 201 g/kg; M: 203 g/kg; H: 198 g/kg) as well as metabolizable energy (**ME**) in grower diets (L: 2,844 kcal/kg; M: 2,820 kcal/kg; H: 2,892 kcal/kg) and finisher diets (L: 3,059 kcal/kg; M: 3,035 kcal/kg; H: 3,035 kcal/kg) were comparable.

### Jejunal Digesta and Fecal Sample Collection

A total of *n* = 75 broilers were considered in this study with an emphasis on d 17, d 24, and d 37. For jejunal digesta collection at d 17 (*n* = 16 from pens), d 24 (*n* = 32 from pens), and d 37 (*n* = 27 from single cages), birds were anesthetized by electrical stunning and slaughtered by exsanguination. Whole jejunum tissue (approx. 5 cm) was collected proximal to the Merkel's diverticulum and cut lengthwise to sample the intestinal digesta gently with a spatula. Digesta were snap-frozen in liquid nitrogen and stored at −80°C until DNA isolation. Additionally, deposited fecal samples from a period of 4 h were collected from birds housed in single cages at d 17, d 24, and d 37. Fecal samples were stored at −20°C until further analysis.

### DNA Isolation, 16S rRNA Gene Amplicon Sequencing and Bioinformatics Analysis

Microbial DNA was isolated from the broiler digesta samples using the DNeasy PowerLyzer PowerSoil Kit (QIAGEN, Hilden, Germany) according to the manufacturer's guidelines. The samples were incubated at 70°C and 95°C for 10 min each before bead-beating with Precellys 24 homogenizer (PEQLab Biotechnology GmbH, Darmstadt, Germany). DNA concentration was determined using the NanoDrop ND-2000 spectrophotometer (Thermo Fisher Scientific, Dreieich, Germany). Amplicons of the 16S rRNA gene were synthesized in duplicates using primers specific to the V4 (515F:GTGCCAGCMGCCGCGGTAA and 806R: GGACTACHVGGGTWTCTAAT) hypervariable region alongside adapters and barcodes (Hugerth et al., 2014). The polymerase chain reaction was performed with the GoTaq G2 Hot Start Master Mix (Promega, Walldorf, Germany), with temperature, timing, and cycle regimen set as follows: initial denaturation step at 95°C for 2 min, 35 cycles, denaturation at 95°C for 30 s, annealing at 50°C for 60 s and 72°C for 90 s, and a final extension at 72°C for 10 min. Amplicons were prepared in duplicates, combined, purified, and normalized using a SequalPrep normalization plate (Thermo Fisher Scientific, Darmstadt, Germany). Afterward, libraries were sequenced on a HiSeq 2500 instrument (Illumina, San Diego, CA). After demultiplexing, raw data were analyzed with the mothur software (version 1.44.1) (Schloss et al., 2009). The Silva reference database (release 138) was employed for the global alignment of the 22,942,877 sequence reads, after which annotated operational taxonomic units (**OTUs**) were retrieved at 97% sequence identity.

### Fecal Mineral Content and Phytate Measurement

The freeze-dried fecal samples were weighed, milled, and digested via microwave treatment to solve the analytes and obtain an effective yield. Total P and calcium (**Ca**) content was ascertained via inductively coupled plasma-optical emission spectroscopy (**ICP-OES**) (UEA Consulting Ltd., Norwich, UK).

For phytate quantification, a total of 100 mg of fecal samples were added to 500 µL deionized water and homogenized on ice. After a centrifugation step for 20 min at 10,000 rpm at 4°C, the supernatant was stored at −20°C and used for further analyses. The fecal phytate content was analyzed in a microplate format via a colorimetric assay (orb707384, Biorbyt, Cambridge, UK).

### Statistical Data Analysis

The broiler chicken's daily P and Ca intake was calculated based on feed intake and the corresponding dietary composition for the respective periods as presented previously (Omotoso et al., 2023). These parameters and fecal P, Ca, and phytate were analyzed at each phase using a linear model: γ_ij_ = μ + d_i_ + s_j_ + ε_ij_, where γ_ij_ are the measurements of the response variable (i.e., zootechnical traits, fecal minerals), μ represents the overall mean, d_i_ represents effect of the dietary P group, s_j_ represents sex effect, and ε_ij_ represents the residual error. Analyses were performed using the R package stats and lmerTest (Kuznetsova et al., 2017; R Core Team, 2023). The pairwise comparison of means between dietary groups was achieved with the embedded Tukey post hoc test. Microbial alpha diversity parameters including the Shannon diversity index, inverse Simpson index, and species richness using the abundance-based coverage estimator (**ACE**) were analyzed to ascertain the jejunal microbiota richness, evenness, and diversity in response to the dietary P using vegan package v2.5-7 and phyloseq v1.42.0 embedded in R (R Core Team, 2023). Furthermore, the non-metric multidimensional scaling (**NMDS**) ordination was visualized based on the Bray-Curtis dissimilarities and the similarities were checked with the analysis of similarities (**ANOSIM**) approach in vegan package v2.5-7 embedded in R (R Core Team, 2023). The relative abundance of the microbiota was visualized using taxa plot at the genus level employing the R software (R Core Team, 2023). Differences were considered as statistically significant at *P* ≤ 0.05. After subsampling the 16S data for each sample to 96,873 reads, dietary effects on microbial abundance were assessed at the genus level for samples collected at d 24 and d 37 using Wald test statistics embedded within the DESeq2 in R platform (DOI:10.18129/B9.bioc.DESeq2). At least 30 counts in more than 6 individuals were used as filtering criteria at the genera level. Genera with a Benjamini-Hochberg-adjusted *P* value <0.05 were considered as statistically significant.

## RESULTS

The current study investigated the role of the broiler's jejunal microbiota in adapting and contributing to maintain the host's nutrient efficiency following a P depletion strategy.

### Mineral Intake of Total Phosphorus and Calcium

The early grower developmental phase (d 10–17) revealed significant differences between the dietary intake of P between the M and H groups, indicating that the feed formulations were effective (Table 1). Following the dietary P depletion (d 17–24), significantly lower levels were observed for P intake in the depleted groups (ML, HL) compared to non-depleted groups (i.e., recommended, MM and high, HH). Moreover, total P intake was higher in HH animals compared to MM animals. The total Ca intake was significantly reduced in HL compared to HH animals. At d 25 to 37, total P intake was significantly reduced in the depleted groups (MLL, HLL) compared to the respective non-depleted animals (MHH, HHH). Moreover, total P and calcium intake were higher in HHH animals compared to MMM animals at this growth phase (d 25–37).

**Table 1:** Total phosphorus (P) and calcium (Ca) intake of broilers raised in individual metabolic units and fed divergent amounts of dietary P throughout the developmental phases. Values are displayed as mean ± SEM.

Phase	Diet (*n*)	Total P intake (mg/d)	Ca intake (mg/d)
D 10–17	M (*n* = 16)	418 ± 18^b^	568 ± 25
	H (*n* = 16)	537 ± 21^a^	560 ± 22
D 17–24	ML (*n* = 8)	397 ± 24^c^	803 ± 49^ab^
	HL (*n* = 8)	385 ± 17^c^	779 ± 34^b^
	MM (*n* = 7)	612 ± 28^b^	870 ± 39^ab^
	HH (*n* = 8)	844 ± 45^a^	903 ± 48^a^
D 24–37	MLL (*n* = 7)	572 ± 49^c^	1129 ± 96^b^
	HLL (*n* = 6)	570 ± 83^c^	1125 ± 164^b^
	MMM (*n* = 6)	948 ± 99^b^	1295 ± 131^b^
	HHH (*n* = 8)	1398 ± 49^a^	1450 ± 51^a^

a,b,c Indicate significant differences between groups (*P* < 0.05); L, low P diet; M, medium P diet; H, high P diet. Consecutive letters indicate the dietary treatment in experimental periods.

### Fecal Content of Total Inorganic Phosphorus and Calcium

**Figure 1:**
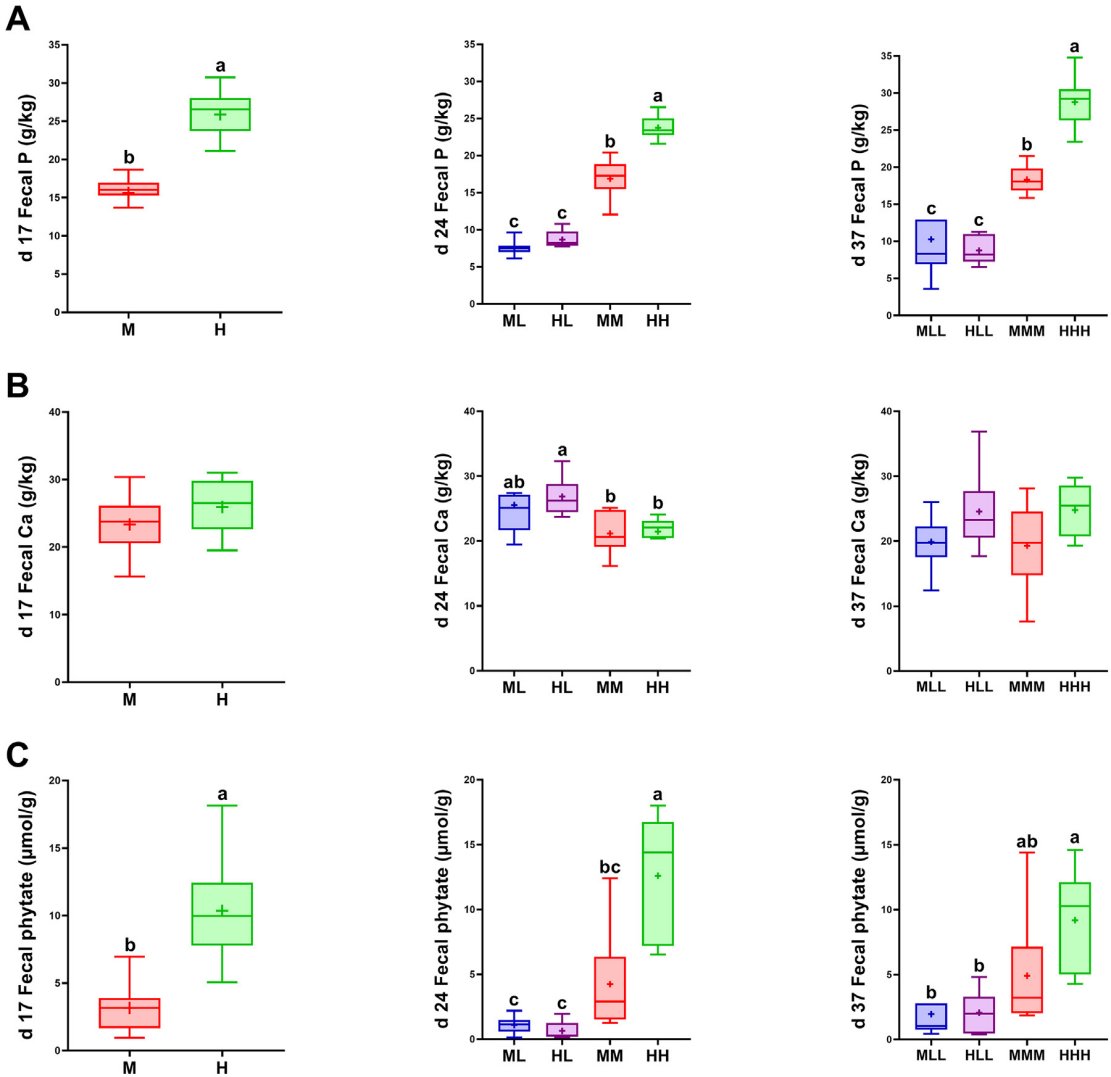
Fecal content of total inorganic phosphorus (A), calcium (B), and phytate (C) of broilers fed varied levels of dietary P at d 17, d 24, and d 37 of life; L, low P diet; M, medium P diet; H, high P diet: Consecutive letters indicate the dietary treatment in each experimental periods. ^a,b,c^Indicate significant differences between groups (*P* < 0.05).

At d 17, the analysis of total mineral P in fecal samples revealed a significantly higher level in H (*n* = 16; 25.87 ± 0.75 g) animals compared to M (*n* = 16; 15.63 ± 0.52 g) animals (Figure 1A). At d 24 and d 37, the P depletion groups ML (*n* = 8; 7.55 ± 0.35 g), HL (*n* = 8; 8.67 ± 0.41 g), MLL (*n* = 7; 8.13 ± 1.24 g), and HLL (*n* = 6; 8.76 ± 0.78 g) showed reduced fecal P levels compared to the respective non-depleted groups MM (*n* = 7; 16.86 ± 1.02 g), HH (*n* = 8; 23.75 ± 0.55 g), MMM (*n* = 6; 18.40 ± 0.96 g), and HHH (*n* = 8; 28.78 ± 1.23 g). Additionally, the comparison of the non-depleted groups revealed significantly higher levels of fecal P in HH and HHH compared to MM and MMM (Figure 1A). The Ca levels in the broilers' feces differed significantly at d 24 with higher levels in the depleted HL group (*n* = 8; 26.85 ± 1.03 g) compared to the corresponding non-depleted HH group (*n* = 8; 21.44 ± 0.85 g) (Figure 1B). Total fecal Ca levels were unaffected by the diets at d 17 and 37 (Figure 1B). At d 17, the analysis of fecal phytate revealed a significantly higher level in H (*n* = 14; 10.36 ± 0.96 µmol/g) animals compared to M (*n* = 13; 3.16 ± 0.48 µmol/g) animals (Figure 1C). At d 24 and d 37, the P depletion groups ML (*n* = 7; 1.09 ± 0.27 µmol/g), HL (*n* = 6; 0.64 ± 0.29 µmol/g), MLL (*n* = 7; 1.96 ± 0.76 µmol/g), and HLL (*n* = 6; 2.07 ± 0.67 µmol/g) showed lower fecal phytate levels compared to the non-depleted groups HH (*n* = 8; 12.61 ± 1.68 µmol/g) and HHH (*n* = 8; 9.19 ± 1.36 µmol/g), but did not differ significantly from groups MM (*n* = 6; 4.24 ± 1.72 µmol/g) and MMM (*n* = 6; 4.92 ± 1.96 µmol/g). Additionally, the comparison of the non-depleted groups revealed significantly higher levels of fecal phytate in HH compared to MM at d 24 (Figure 1C).

### Alpha Diversity Indices of Jejunal Microbiota

**Figure 2:**
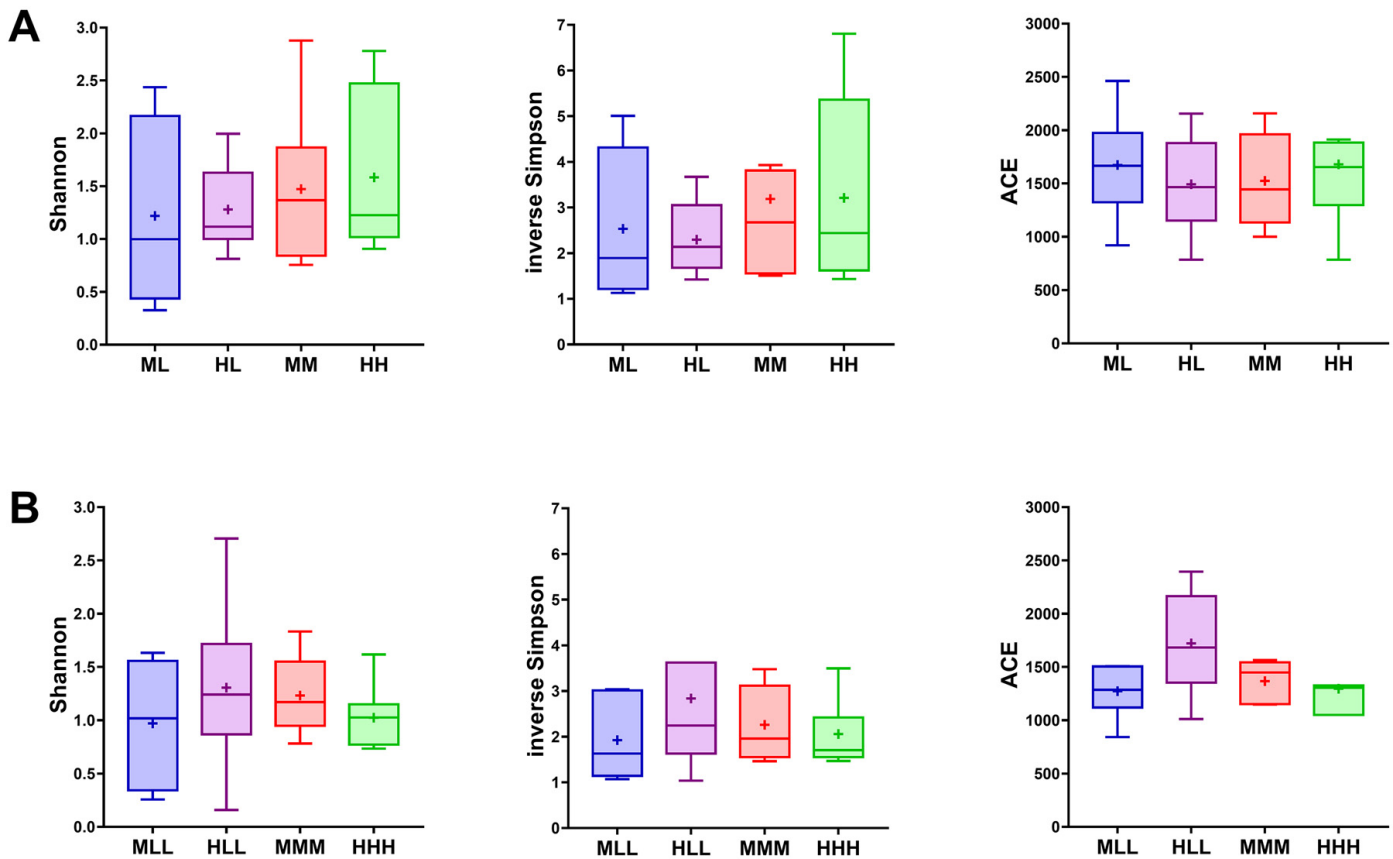
Boxplots showing the alpha diversity of the jejunal microbiota of broilers chickens fed varying amounts of phosphorus (P) at d 24 (A) and d 37 (B). Data based on 16S sequencing were used to calculate the Shannon index, inverse Simpson index and species richness using ACE. ACE, abundance-based coverage estimator; L, low P diet; M, medium P diet; H, high P diet. Consecutive letters indicate the dietary treatment in each experimental period.

To ascertain the response of the jejunal microbiota to the varied P levels fed to the broiler chickens at the grower and finisher developmental phases, alpha diversity indices which account for the distribution or abundance of OTUs within the population were calculated. Shannon, inverse Simpson, and ACE indices revealed no significant differences between the P dietary groups at d 24 and d 37 (Figure 2A and B).

### Composition of Jejunal Microbiota

**Figure 3:**
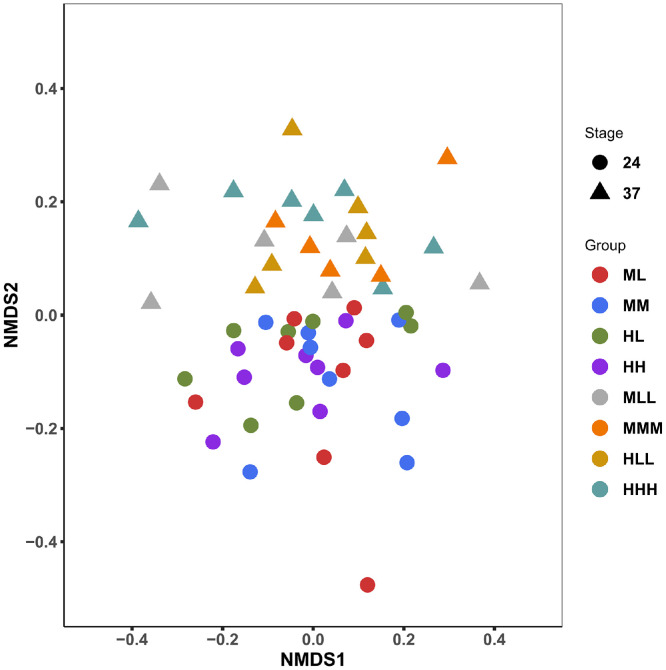
Nonmetric dimensional scaling (NMDS) ordination showing the compositional variation of jejunal microbiota in broilers fed divergent phosphorus (P) diets. Animals were sampled at d 24 (ML, HL, MM, HH) and at d 37 (MLL, HLL, MMM, HHH). L, low P diet; M, medium P diet; H, high P diet. Consecutive letters indicate the dietary treatment in each experimental period.

The non-metric dimensional scaling ordination was used to access the compositional and structural variation of broiler chicken jejunal microbiota. The NMDS analysis revealed an age-based clustering of the jejunal microbial communities (ANOSIM, *r*^2^ = 0.238, *P* = 0.001) with no corresponding influence of the varied P diets within age (*P* > 0.05, Figure 3).

### Relative Abundance of Jejunal Microbiota

**Figure 4:**
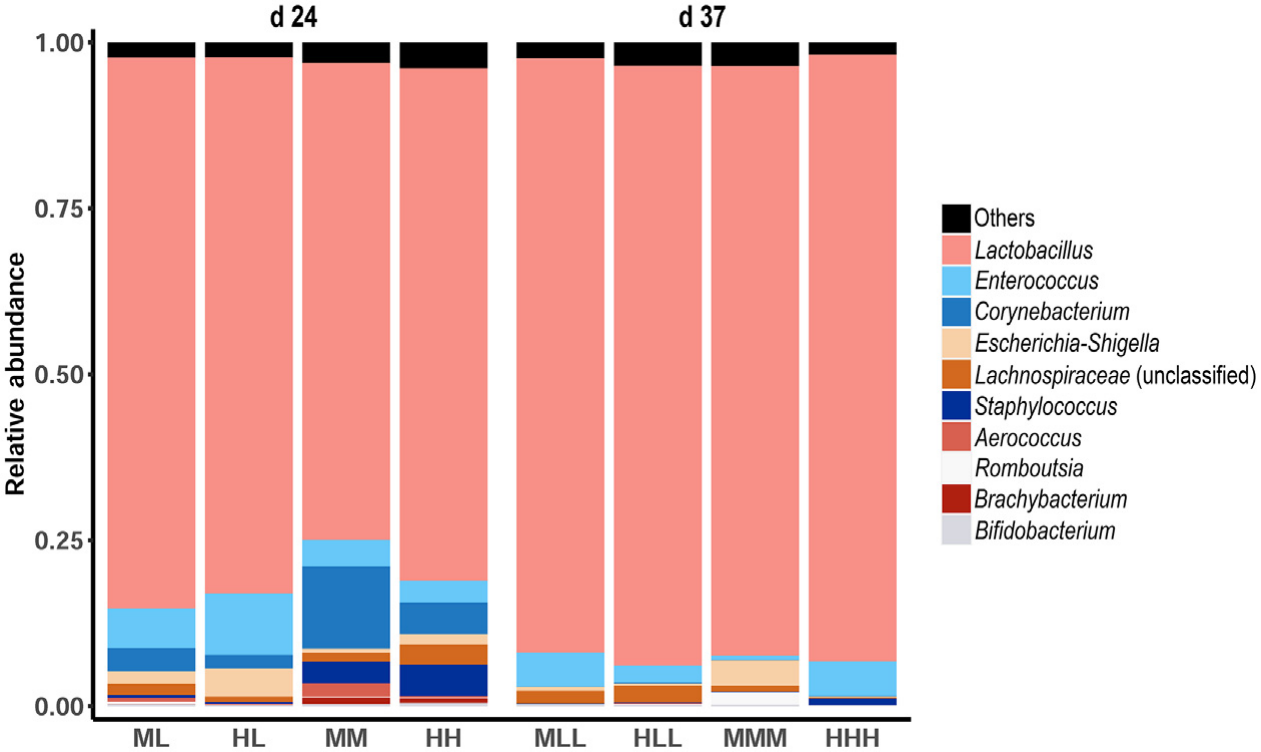
Taxonomic plot showing the 10 predominant relative abundance of the microbial genera in jejunal digesta of broilers fed divergent phosphorus (P) diets. Animals were sampled at d 24 (ML, HL, MM, HH) and at d 37 (MLL, HLL, MMM, HHH). L, low P diet; M, medium P diet; H, high P diet. Consecutive letters indicate the dietary treatment in each of the experimental periods.

Regarding the relative abundance of microbiota in the broilers' jejunum fed varied P diets, visualization using a taxaplot at the genera level identified the *Lactobacillus* of the phylum Firmicutes as the most predominant genus (Figure 4). Further comparisons of relative abundance at genus level between dietary groups revealed 16 and 4 significantly differentially abundant taxa at d 24 and at d 37, respectively (Table 2). At d 24, significantly increased relative abundance of the genera *Facklamia* (HL>HH) was observed in jejunum of broilers fed depleted P diets compared to non-depleted birds. The relative abundances of genera that decreased in the low-P diets compared to the non-depleted diets included *Anaerocolumna* (HL<HH), *Blautia* (HL<HH), *Brachybacterium* (ML<MM; HL<HH), *Brevibacterium* (HL<HH; ML<MM), *Candidatus arthromitus* (HL<HH), *Fusicatenibacter* (HL<HH), *Jeotgalicoccus* (ML<MM), *Lachnoclostridium* (HL<HH), *Monoglobus* (HL<HH), unclassified genera of *Staphylococcaceace* (HL<HH), and *Staphylococcus* (HL<HH; ML<MM). At d 37, a significant differential relative abundance between the depleted and non-depleted P groups was observed in the genera *Romboutsia* (MLL<MMM), while unclassified genera of *Lachnospiraceae* (HLL>HHH) and *Ruminococcaceae* (HLL>HHH) increased in the broilers fed depleted compared to those fed the non-depleted P diets (Table 2). For comparisons of MM and HH diets at d 24, jejunal microbes were observed to be increased in broilers fed the high P diet, including *Blautia* (HH>MM), *Candidatus arthromitus* (HH>MM), *Eisenbergiella* (HH>MM), unclassified genera of *Enterobacteriaceae* (HH>MM), *Monoglobus* (HH>MM), and unclassified genera of *Selenomonadaceae* (HH>MM). In contrast, decreased abundances were found for unclassified genera of *Staphylococcaceae* (MM>HH) and *Aerococcus* (MM>HH). At d 37, a decrease in the differential abundance of microbes was observed between the MMM and HHH diets in *Escherichia-Shigella* (MMM>HHH) and *Romboutsia* (MMM>HHH). The abundance of *Escherichia-Shigella* suggests the possibility of a recent infection episode in individuals of the MMM group.

**Table 2:** Relative abundances of genera in the jejunal digesta collected from broilers fed varied levels of dietary phosphorus (P) at the grower and finisher developmental stages. Listed genera differed significantly (Benjamini-Hochberg-adjusted *P* < 0.05) between dietary groups, with the log2 fold-change expression in a given comparison. Data are presented as mean ± SEM.

D 24	Relative abundance of jejunal microbiota within the dietary P groups (%)				Contrast	
Genera	ML	HL	MM	HH	Fold change (log2)	Adjusted *P* value
*Aerococcus*	0.6 ± 0.360	0.12 ± 0.027	2.04 ± 1.068	0.15 ± 0.082	MM>HH (3.77)	<0.001
*Anaerocolumna*	0.02 ± 0.014	0.003 ± 0.002	0.03 ± 0.016	0.18 ± 0.073	HH>HL (5.55)	<0.001
*Blautia*	0.09 ± 0.047	0.03 ± 0.008	0.11 ± 0.087	0.22 ± 0.120	HH>HL (3.06)	0.010
					HH>MM (3.29)	0.008
*Brachybacterium*	0.02 ± 0.008	0.01 ± 0.005	0.95 ± 0.522	0.7 ± 0.302	HH>HL (5.62)	<0.001
					MM>ML (5.55)	<0.001
*Brevibacterium*	0.003± 0.001	0.002 ± 0.001	0.04 ± 0.026	0.12 ± 0.070	HH>HL (4.90)	<0.001
					MM>ML (3.90)	0.028
*Candidatus arthromitus*	0.02 ± 0.015	0.01 ± 0.003	0.01 ± 0.007	0.14 ± 0.047	HH>HL (5.49)	0.001
					HH>MM (3.46)	0.009
*Eisenbergiella*	0.02 ± 0.010	0.02 ± 0.017	0.01 ± 0.003	0.04 ± 0.022	HH>MM (3.20)	0.044
*Enterobacteriaceae* (uncl.)	0.03 ± 0.019	0.07 ± 0.028	0.02 ± 0.007	0.34 ± 0.273	HH>MM (4.07)	0.001
*Facklamia*	0.04 ± 0.021	0.05 ± 0.016	0.19 ± 0.082	0.004 ± 0.002	HL>HH (3.37)	0.017
					MM>HH (5.28)	0.001
*Fusicatenibacter*	0.05 ± 0.031	0.01 ± 0.005	0.02 ± 0.014	0.11 ± 0.056	HH>HL (3.31)	0.017
*Jeotgalicoccus*	0.05 ± 0.023	0.03 ± 0.014	0.5 ± 0.204	0.03 ± 0.017	MM>HH (3.97)	0.008
					MM>ML (3.32)	0.044
*Lachnoclostridium*	0.02 ± 0.008	0.01 ± 0.006	0.03 ± 0.008	0.09 ± 0.029	HH>HL (2.83)	0.007
*Monoglobus*	0.04 ± 0.018	0.01 ± 0.004	0.02 ± 0.012	0.17 ± 0.074	HH>HL (3.51)	0.008
					HH>MM (3.10)	0.027
*Selenomonadaceae* (uncl.)	0.01 ± 0.007	0.03 ± 0.015	0.001 ± 0.001	0.03 ± 0.030	HH>MM (4.01)	0.008
*Staphylococcaceae* (uncl.)	0.002 ± 0.001	0.002 ± 0.001	0.26 ± 0.150	0.02 ± 0.010	HH>HL (3.87)	0.009
					MM>HH (3.83)	0.008
					MM>ML (6.78)	<0.001
*Staphylococcus*	0.44 ± 0.266	0.22 ± 0.064	3.3 ± 1.872	4.83 ± 2.538	HH>HL (4.46)	<0.001
					MM>ML (2.93)	0.039

D 37	Relative abundance of jejunal microbiota within the dietary P groups (%)				Contrast	
Genera	MLL	HLL	MMM	HHH	Fold change (log2)	Adjusted *P* value

*Escherichia-Shigella*	0.61 ± 0.185	0.28 ± 0.092	3.81 ± 3.658	0.12 ± 0.035	MMM>HHH (4.94)	0.001
*Lachnospiraceae* (uncl.)	1.87 ± 1.673	2.55 ± 1.890	0.91 ± 0.522	0.18 ± 0.051	HLL>HHH (3.86)	0.010
*Romboutsia*	0.04 ± 0.017	0.11 ± 0.102	1.86 ± 1.789	0.04 ± 0.015	MMM>HHH (5.70)	0.002
					MMM>MLL (5.45)	0.010
*Ruminococcaceae* (uncl.)	0.34 ± 0.303	0.53 ± 0.446	0.10 ± 0.067	0.03 ± 0.014	HLL>HHH (4.44)	0.010

L, low P diet; M, medium P diet; H, high P diet. Consecutive letters indicate the dietary treatment in each experimental period.

## DISCUSSION

To mitigate the immediate concerns associated with the environmental loading of P from broiler husbandry (Maguire et al., 2005), studies have investigated effects of moderate dietary P depletion initiated at an earlier growth phase and subsequently repleted with advancing ages for mineral efficiency (Valable et al., 2018; Baradaran et al., 2021). Indeed, our recent analyses on the physiological response of modern high-performance broiler lines challenged with depleted P diets revealed intrinsic mechanisms, spanning hormones, bone traits, as well as intestinal and renal P transporters to maintain P turnover (Omotoso et al., 2023). Subsequently, the resultant fecal P levels mirrored the dietary P intake in the depleted, recommended, and high P feeding groups. A previous study found that broilers fed a balanced Ca:nPP ratio of 2:1 (i.e., 6 g/kg and 3 g/kg DM) had the lowest fecal P excretion, while higher nPP levels at a ratio of 1.33:1 (i.e., 6.0 g/kg and 4.5 g/kg DM) resulted in increased fecal P content (Rama Rao et al., 2006). It was observed in the current study that the birds fed the HL diet had higher levels of fecal Ca at d 24 in comparison to the non-depleted cohorts. Nevertheless, previous findings on broiler chickens fed reduced inorganic P showed increased calcitriol in the blood and intestinal Ca-binding protein levels leading to improved Ca absorption (Friedlander et al., 1977; Wasserman et al., 1992). In fact, serum Ca levels were elevated in both ML and HL compared with the non-depleted groups (Omotoso et al., 2023). In parallel, the analyses revealed evidence of increased bone resorption in the depleted broiler chickens compared with the non-depleted groups. This could indicate increased mineral mobilization to balance P requirements as a consequence of the applied depletion strategy, with excess Ca being excreted accordingly. Comprehensive studies concluded that current recommendations for total Ca content in broiler feed formulations might be overestimated (David et al., 2023), affecting P absorption rates (Selle et al., 2009). In the current study, the resultant fecal P levels were significantly higher in the broilers fed the high P diets than those fed recommended P levels throughout the grower and finisher periods. Higher P excretion rates due to mineral P supplements above recommendations have also been demonstrated in other monogastric species such as pigs (Reyer et al., 2021), with no added benefit observed for bone mineralization (Gerlinger et al., 2021). Thus, the results indicate that broilers fed the high P diet received mineral fractions that exceeded their metabolic demands for growth or maintenance with no additional benefit for the measured traits but resulted in unnecessary fecal losses as reported elsewhere (Rodehutscord et al., 2012; Li et al., 2017).

The gut microbiota has been associated with crucial homeostatic and metabolic processes, which entail, for example, the hydrolysis of phytate in the broiler's GIT, culminating in the host's productivity and welfare (Rinttila and Apajalahti, 2013; Li et al., 2016). In the current study, the overall microbial diversity represented by alpha diversity indices revealed no alterations based on dietary P depletion. Based on the microbial dissimilarity analysis, an age-dependent separation of profiles was observed. In accordance, previous studies have reported clear effects of age on the microbial community that colonizes the broiler's GIT (De Cesare et al., 2019; Zhou et al., 2021). Furthermore, the dominance of the *Lactobacillus* in the current study was consistent with several previously reported studies (Borda-Molina et al., 2016; Kers et al., 2018; Künzel et al., 2021), where *Lactobacillus* presence in the gut accounted for up to 99% of the microbiota fraction. Functionally, the abundance of *Lactobacillus* in the gut has been positively correlated with beneficial functions, including improved gut physiology and increased body weight gain in the chicken (Lokapirnasari et al., 2019; Zhang et al., 2022). More so, it is inferable that the prevalence of *Lactobacillus* might indicate a low complexity of jejunal microbiota in broiler chickens.

The analysis of microbial abundances, particularly the fact that hardly any genera increase in abundance following a P depletion, suggests that shifts in the jejunal microbiota make only a minor contribution to maintaining P homeostasis in broilers. Nevertheless, it remains conceivable that transcriptional changes of the abundant microbiota are affecting mineral metabolism, which would need to be tested by metatranscriptomic approaches.

The phytate profile reported in the current work serves as proxy for intestinal microbiota activity. Results show significantly lowered concentrations in birds fed medium P than in birds fed high P levels at d 17 and d 24, indicating hydrolysis of phosphoric ester forms mediated by a phytase that, based on current knowledge, is likely secreted by the intestinal microbiota in broiler chickens. Indeed, phytase activity of microbiota has been mainly observed in the lower part of the intestinal tract such as the caecum (Dersjant-Li et al., 2015). The observed shifts in fecal phytate content matches previous studies (Shastak et al., 2014) and suggests efficiency mechanisms already at early age. Results further suggest that birds fed currently recommended P levels (M diets) mobilize P from plant sources. The P-depleted groups exhibited a nearly maximal degradation of phytate at d 24 and d 37, providing evidence for intestinal dephosphorylation of phytate to meet metabolic demands. However, to what extent the P mobilized from intestinal phytate is available to the host or the microbiota remains further clarification. According to previous work, P mobilization from intestinal inositol phosphates can be assumed to be incomplete as lower phosphoric ester forms, that is, the degradation products of phytate, are present in feces at varying amounts (Gautier et al., 2018). A study in piglets identified microbial taxa that were positively or negatively correlated to intestinal P levels in terms of proliferation (Reyer et al., 2021). In the current study, exclusively the genus *Facklamia* exhibited an increased abundance in the broiler cohort fed the HL compared to HH diets at d 24. The abundance of *Facklamia* was previously reported to be related with housing and litter management, that is, with higher abundances in fresh litter compared to reused litter material (Wang et al., 2016; Song et al., 2022). The information on its role in relation to nutrient metabolism in the broiler is absent. For certain species of *Facklamia*, enzymatic profiles revealed activity for alkaline phosphatases, while acid phosphatase activities were not observed (Lawson et al., 1999). At d 37, an incremental shift in the gut of broilers fed the HLL diet compared to those that received HHH was observed for unclassified genera belonging to families *Lachnospiraceae* and *Ruminococcaceae*. Broadly, both the *Lachnospiraceae* and *Ruminococcaceae* microbiota families are categorized as beneficial in the human GIT and have been implicated in the fermentation of carbohydrates (Duncan et al., 2007), coupled with the degradation of resistant polysaccharides, for example, starch and cellulose, facilitating digestion of plant-based diets (Collier et al., 2008). The identified taxa may be of interest in further studies to reshape the microbial composition for improved nutrient utilization from dietary P sources.

In contrast to the microbes whose abundance increased after the P depletion diet, *Brachybacterium, Brevibacterium*, and genera of the *Staphylococcaceae* family showed significantly lower abundances in the jejunum, which was consistently observed in both P depletion groups compared to the respective non-depleted controls on d 24. Apparently, these microbial families rely on an intestinal milieu with higher available P content, implying overgrowth of these taxa when P resources are scarce. In this context, an increased intestinal abundance of *Brachybacterium* has been reported from patients with disturbed mineral balance based on chronic kidney disease (Vaziri et al., 2013). Several species of *Brevibacterium* were described as phosphate-accumulating probiotics, which might be more prevalent in high P supply (Anand et al., 2019). Indeed, some taxa show a dominant growth pattern whereby the microbial community structures are subject to dynamics in their composition with corresponding effects on the ability to respond to abiotic and biotic factors (Niccum et al., 2020). The results suggest that the increased proliferation of these mentioned microbial taxa due to high P supply could be considered a biomarker of excessive P intake in commercial broiler chickens.

At the same time, several other taxa were shown here to be generally responsive to the divergent P supply in the different diets. The microbial profiles may support the current hypothesis that an increase in intestinal P levels in mammals stimulates microbial short-chain fatty acid (**SCFA**) production (Heyer et al., 2015). This is consistent with a study in broilers in which a decrease in SCFA, DL-lactate, and acetic acid in the ileum was observed following low P and low Ca diets and subsequently, an increase in these parameters was observed after phytase supplementation (Ptak et al., 2015). This agrees with in vitro studies on the fermentation activity of rumen bacteria, which identified an association between depleted P levels and a reduction in SCFA and bacterial ATP production (Komisarczuk et al., 1987). Moreover, a recent study in chickens reported that dietary P deficiency resulted in decreased SCFA production due to reduced cellulose fermentation, suggesting that intestinal P content modulates the abundance of fibrolytic bacteria (Li et al., 2022).

A body of literature supports some of the observed genera in the context of intestinal SCFA production and P utilization in poultry. Among such taxa are the *Blautia, Anaerocolumna, Candidatus arthromitus*, and unclassified genera of *Selenomonadaceae*, observed to increase significantly in broilers fed the high P diet. The *Blautia*, an anaerobic bacteria specie which clusters into the *Clostridium XIVa* group, became of particular interest in human microbiomics since the knowledge of its ability to synthesize SCFA (Blaak et al., 2020; Nishiwaki et al., 2022). Similarly, *Anaerocolumna*, a major taxonomic microbial group in the gut, is reported to mediate the fermentation of complex polysaccharides and degrading highly lignified diets in polygastrics (Shabana et al., 2020). Furthermore, a previous study in piglets reported that the intestinal abundance of *Selenomonadaceae* genera negatively correlated with P intake, serum P levels and degradation of phytate as well as inositol-5 phosphate in distal parts of the gastrointestinal tract (Reyer et al., 2021). A study on quail (*Cortunix japonica*) reported a positive correlation between the abundance of genera *Candidatus arthromitus* and performance and P utilization traits (Vollmar et al., 2020). However, following a contrasting observation in the current broiler study, wherein *Candidatus arthromitus* was more abundant in the high P group, it is not yet clear whether the abundance of this taxon depends on dietary P levels or the abundance of *Candidatus arthromitus* improves intestinal P mobilization. It should be noted that due to taxonomic reclassifications, sequences assigned to *Candidatus Arthromitus* in vertebrates should be considered as *Candidatus Savagella* (Thompson et al., 2012).

## CONCLUSIONS

In summary, the study showed that P intake, fecal P, and fecal phytate contents are parallel, that is, the P-depleted cohorts excreted less P compared to the non-depleted feeding groups and vice versa. The improved phytate degradation in P-depleted broilers efficiently mobilizes phosphorus from plant feed components, which is also largely observed in the groups fed the currently recommended P supply, while higher P-fed groups excrete substantial amounts of unutilized phytate. However, in addition to the distinct mechanisms for improved P utilization, the analysis of the jejunal microbiota shows only a minor shift in microbial taxa between the P-depleted and non-depleted groups. Furthermore, some microbial taxa proliferated in a P-enriched environment and might be considered as biomarkers of excessive P supply in commercial broiler chicken as well as significant SCFA producers. The microbial composition in jejunum made only a minor contribution to the birds' compensatory mechanism for adaptation following P depletion.

## Appendix Supplementary materials

Image, application 1

## Footnotes

Supplementary material associated with this article can be found in the online version at doi:10.1016/j.psj.2023.103096.
