# Supplementary material for: Jejunal microbiota of broilers fed varying levels of mineral phosphorus
Source: Poult Sci. 2023 Sep 9;102(12):103096. doi: 10.1016/j.psj.2023.103096 (PMC10562922; doi:10.1016/j.psj.2023.103096)
Supplement: Supplementary file 1 [file mmc1.docx]

**Supplementary Table 1: Composition of the experimental diets for broiler chickens at starter, grower, and finisher phases.**

|  |  | Starter (d 1-10) | |  | Grower (d 11-24) | | |  | Finisher (d 25-37) | | |
| --- | --- | --- | --- | --- | --- | --- | --- | --- | --- | --- | --- |
| Ingredient | **Unit** | **100% nPP** | **+50% nPP** |  | **-50% nPP** | **100% nPP** | **+50% nPP** |  | **-50% nPP** | **100% nPP** | **+50% nPP** |
| Wheat | % | 30.0 | 30.0 |  | 32.0 | 32.0 | 32.0 |  | 33.5 | 33.5 | 33.5 |
| Soybean meal (44 % CP) | % | 26.0 | 26.0 |  | 27.0 | 27.0 | 27.0 |  | 24.0 | 24.0 | 24.0 |
| Corn, pre-treated^1^ | % | 19.4 | 19.4 |  | 21.0 | 21.0 | 21.0 |  | 24.0 | 24.0 | 24.0 |
| Soybean concentrate (64 % CP) | % | 11.0 | 11.0 |  | 6.5 | 6.5 | 6.5 |  | 5.0 | 5.0 | 5.0 |
| Soybean oil | % | 5.4 | 5.4 |  | 6.3 | 6.3 | 6.3 |  | 6.3 | 6.3 | 6.3 |
| Calcium carbonate | % | 1.16 | 0.63 |  | 1.37 | 0.94 | 0.48 |  | 1.28 | 0.87 | 0.40 |
| Cellulose powder | % | 0.60 | - |  | 1.10 | 0.50 | - |  | 1.10 | 0.60 | - |
| Corn starch, pre-gelatinized | % | 1.1172 | 1.0472 |  | 0.6573 | 0.7173 | 0.6273 |  | 0.8674 | 0.8374 | 0.8874 |
| Brewer's dried yeast | % | 1.0 | 1.0 |  | 1.0 | 1.0 | 1.0 |  | 1.0 | 1.0 | 1.0 |
| Vitamin & trace element premix^2^ | % | 1.0 | 1.0 |  | 1.0 | 1.0 | 1.0 |  | 1.0 | 1.0 | 1.0 |
| Monocalcium phosphate, (23 % P) | % | 1.60 | 2.80 |  | 0.33 | 1.30 | 2.35 |  | 0.27 | 1.21 | 2.23 |
| Salt, NaCl | % | 0.43 | 0.43 |  | 0.43 | 0.43 | 0.43 |  | 0.43 | 0.43 | 0.43 |
| Choline Cl (50%) | % | 0.39 | 0.39 |  | 0.37 | 0.37 | 0.37 |  | 0.35 | 0.35 | 0.35 |
| DL-Methionine | % | 0.35 | 0.35 |  | 0.34 | 0.34 | 0.34 |  | 0.29 | 0.29 | 0.29 |
| Calcium propionate | % | 0.3 | 0.3 |  | 0.3 | 0.3 | 0.3 |  | 0.3 | 0.3 | 0.3 |
| Lysine HCl | % | 0.21 | 0.21 |  | 0.21 | 0.21 | 0.21 |  | 0.22 | 0.22 | 0.22 |
| Manganese sulphate (33 % Mn) | % | 0.028 | 0.028 |  | 0.028 | 0.028 | 0.028 |  | 0.028 | 0.028 | 0.028 |
| Zinc sulphate (36 % Zn) | % | 0.01 | 0.01 |  | 0.01 | 0.01 | 0.01 |  | 0.01 | 0.01 | 0.01 |
| Copper sulphate (24 % Cu) | % | 0.004 | 0.004 |  | 0.004 | 0.004 | 0.004 |  | 0.004 | 0.004 | 0.004 |
| Vitamin D3 (500.000 IU/g) | % | 0.00078 | 0.00078 |  | 0.0007 | 0.0007 | 0.0007 |  | 0.0006 | 0.0006 | 0.0006 |
| Threonine | % | - | - |  | 0.05 | 0.05 | 0.05 |  | 0.05 | 0.05 | 0.05 |
| ME | kcal/kg | 2,988 | 2,988 |  | 3,059 | 3,059 | 3,059 |  | 3,107 | 3,107 | 3,107 |
| Sum | % | 100 | 100 |  | 100 | 100 | 100 |  | 100 | 100 | 100 |
| Calcium | % | 1.04 | 1.04 |  | 0.90 | 0.90 | 0.90 |  | 0.84 | 0.84 | 0.84 |
| Phosphorus, total | % | 0.78 | 1.05 |  | 0.47 | 0.69 | 0.93 |  | 0.44 | 0.65 | 0.88 |
| Phosphorus, nPP^3^ | % | 0.52 | 0.78 |  | 0.23 | 0.45 | 0.68 |  | 0.21 | 0.42 | 0.64 |

^1^Corn, pre-treated – hydrothermal treatment

^2^Vitamin & trace element premix (SNIFF Spezialdiäten GmbH, Soest, Germany) provided per kg of feed: vitamin A (retinyl acetate), 15.000 IE; vitamin D3 (cholecalciferol), 1.100 IE; vitamin E (all-rac-alpha-tocopheryl acetate), 100 mg; vitamin K3 (menadione), 7 mg; Fe (as FeSO4), 100 mg; Zn (as ZnSO4), 50 mg; Mn (as MnSO4), 30 mg; Cu (as CuSO4), 5 mg; Se (as Na2SeO3), 0.1 mg; I (as Ca(IO3)2), 2.0 mg;

^3^nPP – non-phytate phosphorus
